# Comprehensive prognostic and immunological analysis of Cullin2 in pan-cancer and its identification in hepatocellular carcinoma

**DOI:** 10.18632/aging.205848

**Published:** 2024-05-22

**Authors:** Longmei Jia, Xiaoqiang Zhang, Tao Zhou, Jinyan Xie, Jiejing Jin, Dandan Zhang, Chao Zhu, Rong Wan

**Affiliations:** 1 Department of Nuclear Medicine, The Second Affiliated Hospital of Nanchang University, Nanchang 330006, Jiangxi, China; 2 Department of Thoracic Surgery, The Second Affiliated Hospital of Nanchang University, Nanchang 330006, Jiangxi, China; 3 Jiangxi Key Laboratory of Molecular Medicine, Nanchang 330006, Jiangxi, China; 4 Department of Genetic Medicine, The Second Affiliated Hospital of Nanchang University, Nanchang 330006, Jiangxi, China; 5 Department of General Surgery, The Second Affiliated Hospital of Nanchang University, Nanchang 330006, Jiangxi, China; 6 Department of Pharmacology, Nanjing Medical University, Nanjing 211166, Jiangsu, China

**Keywords:** CUL2, pan-cancer, prognosis biomarker, immunological analysis, HCC

## Abstract

Background: As a member of the Cullin family, Cullin2 (CUL2) is involved in the development and spread of different types of cancers. However, the precise role of CUL2 in human cancer remains largely elusive.

Methods: In this study, various databases were applied to observe the CUL2 expression. Kaplan-Meier and Spearman correlation analyses were employed to investigate the potential links between CUL2 level, patient prognosis, and the infiltration of immune cells. In addition, the association between CUL2 and the efficacy of immunotherapy in an immunotherapy cohort was investigated. Moreover, the expression and distribution of CUL2 in cells were observed using the Human Protein Atlas (THPA) database. Finally, clinical tissue specimens and *in vitro* function assays were conducted to validate the expressions and effects of CUL2 on the biological functions in hepatocellular carcinoma (HCC) cells.

Results: While there are variations in CUL2 expression across different organs and cell types, it is notably upregulated in a majority of tumor tissues. In addition, CUL2 gene mutations are common in multiple cancers with low mutation rates and CUL2 is closely related to the prognosis of some cancer’s patients, some immune regulatory factors, TMB, MSI, MMR genes, and DNA methylation. Further, our results found that downregulating CUL2 inhibits the proliferation, and migration abilities.

Conclusions: The expression of CUL2 has an impact on the prognosis of various tumors, and this correlation is particularly noteworthy due to its significant association with the infiltration of immune cells within tumors. CUL2 was an oncogene contributing to the progression of HCC.

## INTRODUCTION

Tumors are diseases in which cell growth is abnormal. Normal cell growth regulation mechanisms cannot control tumor cells and are often considered multigene conditions [1, 2]. The Cullin protein family is vital for facilitating ubiquitin-mediated proteolysis, a mechanism that controls protein levels and adjusts cellular conditions to facilitate stress adaptation [3]. Studies have pointed out that Cullin proteins lack oncogenic or tumor suppressor properties. Their impact on oncogenesis is influenced by their interaction with substrates and receptors, and mutations in these proteins lead to the buildup of oncoproteins [4]. Playing a crucial role, CUL2 acts as a significant E3 ligase within the ubiquitin-proteasome pathway [5]. It is engaged in the regulation of vital cellular functions, including protein degradation and the advancement of the cell cycle [6].

Recent research indicates that CUL2 plays a significant role in the initiation and progression of tumors. Deviant CUL2 expression can impact cancer prognoses and is intricately linked to tumor immune infiltration. For example, CUL2 is overexpressed in many cancers, such as pancreatic ductal adenocarcinoma [7], hepatocellular carcinoma [8], esophageal cancer [9], and cervical cancer [10], and promotes liver fibrosis [11]. CUL2 positivity was associated with a significantly worse prognosis in high-risk carcinoma but not in low-risk carcinoma, according to Shipitsin et al. [12] In addition, CUL2 can also affect the sensitivity of tumor cells to chemotherapy and immunotherapy [13]. Clinical trials have shown that inhibitors of ubiquitin ligase enhance the mortality of neoplastic cells and heighten their vulnerability to chemotherapy and radiotherapy [3]. Hence, delving deeper into the functions and mechanisms of CUL2 in tumor formation and advancement holds considerable importance in evaluating prognoses and devising effective cancer treatments.

Pan-cancer analysis has gained growing attention with the continuous advancement of cross-cancer and multigroup data. It has enabled a more thorough comprehension of the etiology of malignancies. Given the intricate nature of tumorigenesis, it is crucial to investigate the expression of genes of interest and evaluate their potential molecular mechanisms and correlation with clinical prognosis. In total, a comprehensive analysis of cancer datasets was taken to create gene expression profiles for CUL2 in diverse cancer types. Additionally, we explored the connections between CUL2 expression and factors such as prognosis, enriched gene sets, immune cell infiltration, and expression of immunomodulators. Drawing from these discoveries, we propose CUL2 as a novel prognostic marker and indicator of immunotherapy effectiveness in cancer. Our findings offer potential guidance for future research directions concerning CUL2.

## MATERIALS AND METHODS

### Acquisition and processing of public data

To conduct a comprehensive pan-cancer analysis covering 33 cancer types, RNA-sequencing data from the UCEC database were downloaded [14]. This database integrated the TCGA database and GETx Project, providing a wealth of high-quality sequencing data for our analysis. The downloaded datasets were processed and normalized to ensure consistency and quality. Specifically, the Fragments Per Kilobase per Million [FPKM]+1 values were batched and transformed to log2 scale, a widely used normalization method in RNA-Seq analysis. Out of the 33 cancer types, 22 of them had corresponding normal tissue data available, which was obtained by utilizing the matching information provided by the Gene Expression Profiling Interactive Analysis 2 website [15]. Supplementary Table 1 contains the cancer abbreviations.

### Gene mutation landscape, immunohistochemistry (IHC) and immunofluorescence (IF) analysis

The cBioPortal database was used to explore the CUL2 mutation information including alteration frequency, mutation type, and copy number alteration [16]. In addition, we used the Sengerbox website to investigate the expression data of CUL2 and MMR genes as DNA methylation markers in pan-cancer. The expression in both liver cancer and adjacent tissues and the distribution in various cell lines, including A-431, U-251MG, and PC-3 cells of CUL2 were examined using the Human Protein Atlas website public database [17].

### Prognosis analysis of CUL2 and GSEA

Clinical data and prognostic information for pan-cancer cases were sourced from the UCEC database, originating from a TCGA study focused on prognosis [18]. The dataset included OS, PFI, and DSS. We employed the survival and survminer R packages, employing the Log-rank test to assess differences between high and low-expression subgroups. Statistical significance was indicated by a p-value below 0.05. We used the GSEA computational method to investigate the impact of CUL2 expression on cancer by analyzing CUL2 enrichment. To achieve this, we obtained the “gmt” file of the hallmark gene set, from the Molecular Signatures Database website. The GSEA analysis was conducted using the “clusterProfiler” R package, and the results were summarized using a bubble plot created with the “ggplot2” R package [19].

### Immune cell infiltration analysis in TIMER2

We utilized the TIMER (http://timer.cistrome.org/) data source [20], which contains RNA sequencing data from tumor tissues, to generate a heatmap via Spearman correlation analysis. The heatmap was taken to compare CUL2 mRNA with the infiltration levels of different immune cell subtypes. This analysis may provide insights into potential immunotherapeutic strategies for different types of cancer. In addition, we also examined the impact of the tumor microenvironment (TME) on tumor advancement and metastasis, concentrating particularly on the non-tumor constituents [21].

### The relationship between the CUL2 expression and immunotherapy

Treating cancer patients with immunotherapy is an established and integral approach. Immune checkpoint inhibitors (ICIs) have demonstrated impressive potential in treating various types of cancer [22, 23]. Analyzing the expression patterns of immune checkpoint-related genes in either tumor cells or immune cells can effectively predict the clinical benefit of checkpoint inhibitor therapies [24]. Furthermore, numerous studies have highlighted that TMB, MSI, and the emergence of neoantigens due to somatic mutations play pivotal roles in eliciting immune responses against tumors [25]. The TCGA database was used to obtain gene mutation data for all cancer types for this study. We also used the Sangerbox website’s “Tool” module and Spearman’s correlation test to investigate the correlation between CUL2 expression and neoantigens. The results were presented in the form of heat maps or radar plots.

### Cell culture, reagents, and RNA interference

Hepatocellular carcinoma (HCC) cells were sourced from the cell bank at the Chinese Academy of Sciences Shanghai. The cells were Cells were cultured under conventional conditions. The antibodies and reagents were described in Supplementary Table 2. Plasmids encoding *shRNAs* against CUL2 were synthesized by Genepharma Company (Shanghai, China). The target sequences are *shCUL2* (5’-GTCCAGTGGTTTACCTCATAT-3’).

### Western blotting and quantitative real-time PCR (qRT-PCR)

Western blotting and qRT-PCR assays were performed as previously described. The primers used in PCR are CUL2, 5’-ACGACAATAAAAGCCGTGGTC-3’ and 5’-GGATAGGCCACACATAAAGCAT-3’; GAPDH, 5’- GGAGCGAGATCCCTCCAAAAT-3’ and 5’- GGCTGTTGTCATACTTCTCATGG-3’.

### Cell proliferation and transwell assays

For the CCK8 assay, 2,000 transfected cells were seeded in each well of a 96-well plate. They were tested using Cell Counting Kit-8 (CCK-8) according to the manufacturer’s instructions. For the EdU assay, the Cell-Light EdU DNA Cell Proliferation Kit was utilized. The ratio of EdU-positive cells to total cells is used to calculate the proliferation rate. The cells were seeded onto coverslips in a 6-well plate. Reagent A was added to the culture medium at a 1:1000 dilution and incubated for 2 hours before detection. After fixing with 4% paraformaldehyde, cells were stained with a fluorescent dye for 30 minutes, with Hoechst33342 used for nuclear counterstaining. To perform the colony formation assay, shRNA plasmid transfected HCC cells were seeded at a density of 500 cells per well in a 6-well plate. The cells were then cultured for 2 weeks, after which they were treated with 4% paraformaldehyde. The fixed cells were subsequently stained with a 0.5% crystal violet solution. For transwell assays, the specific experimental method is carried out according to the conventional steps.

### Statistical analysis

To conduct bioinformatic analyses, R software (https://www.r-project.org/) was used. The Wilcoxon Rank Sum Test or the Kruskal Wallis Rank Sum Test were used to compare groups. To clarify the correlations between groups, Spearman’s correlation analyses were performed. GraphPad Prism 9.0 software was used to analyze the experimental data. All experiments were carried out in triplicate. The mean ± SD was used to report the results. The differences between groups were examined using the Student’s t-test. All statistical analyses were performed using a two-sided approach, considering statistical significance as p < 0.05.

### Data availability statement

The methodology section describes the databases and techniques used to analyze publicly accessible datasets in this study.

## RESULTS

### Expression analysis of CUL2 across cancers

**Figure 1:**
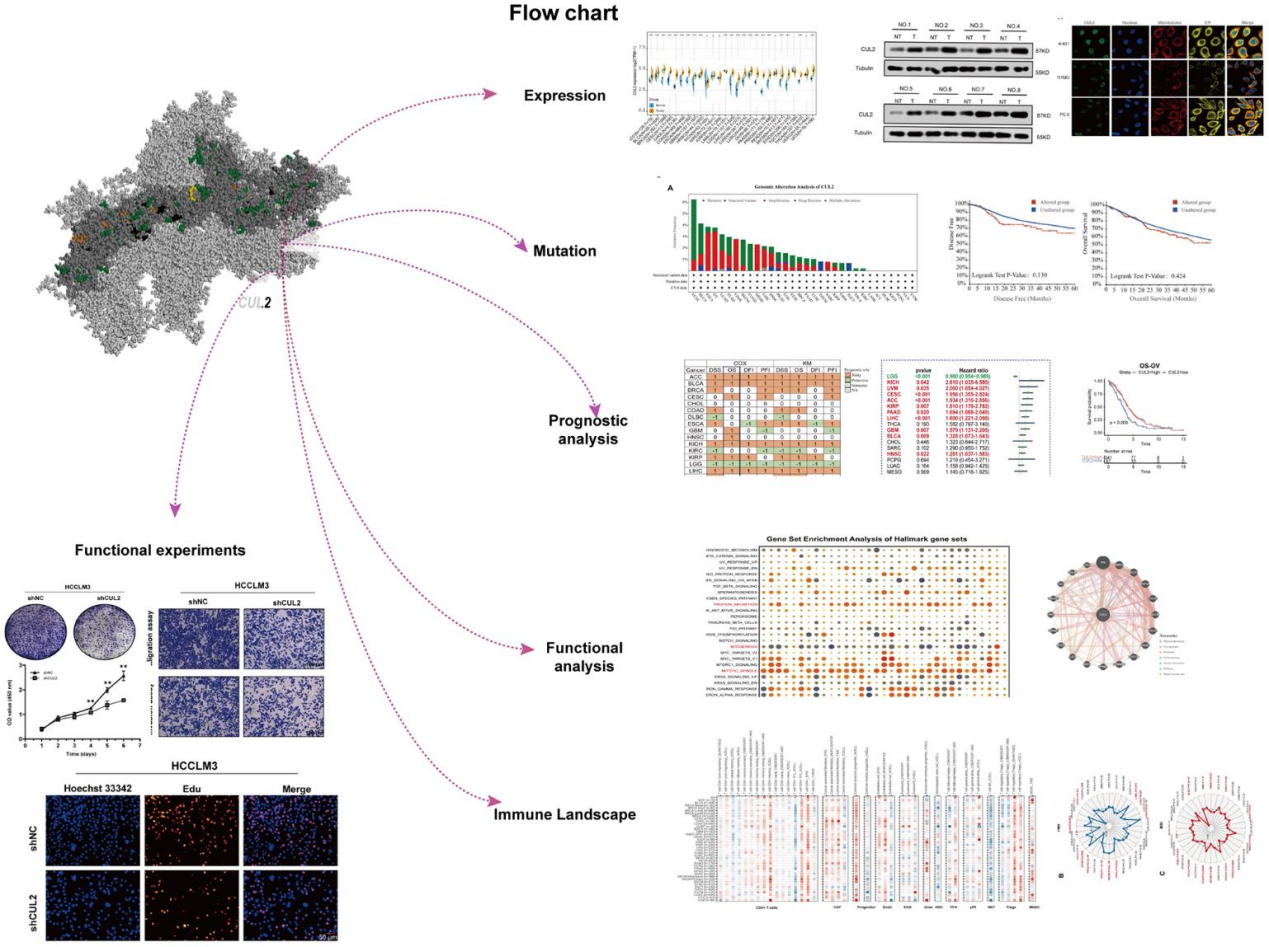
This study’s design and workflow.

**Figure 2:**
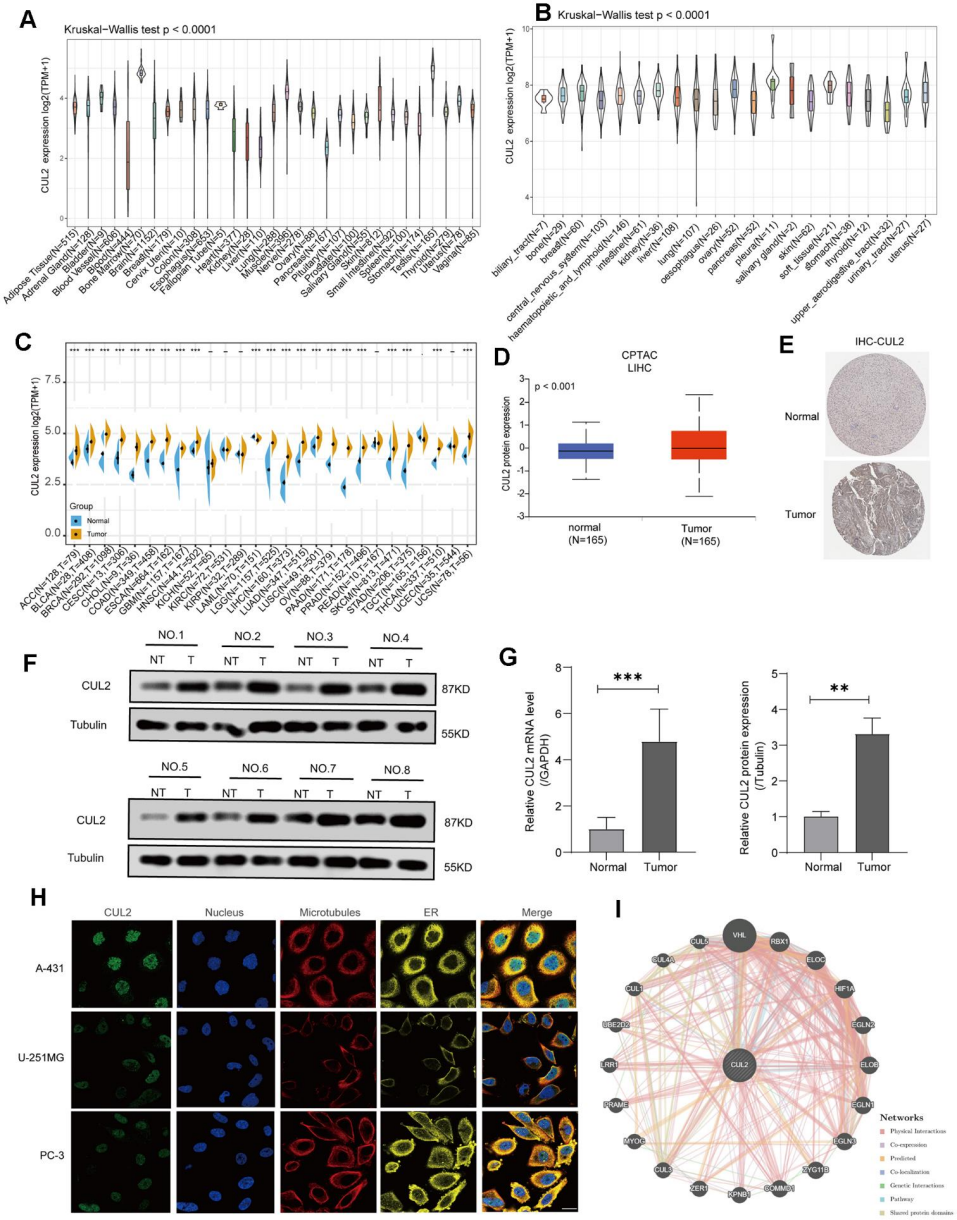
**CUL2 expression analysis in various cancers.** (**A**) The level of CUL2 expression in normal tissues. (**B**) The level of the CUL2 in tumor cell lines. (**C**) A comparative analysis of CUL2 expression levels between various tumors and healthy tissues was conducted using TCGA and GTEx databases data. The box plot data was provided, and Log2 (TPM+1) was used to represent the log scale. (**D**) The CUL2 protein expression in LIHC is based on the CPTAC website. (**E**) Representative images from the HPA database depict immunohistochemical staining analysis of CUL2 in LIHC tissue and adjacent normal tissue are displayed. (**F**, **G**) The protein and mRNA levels of CUL2 were analyzed via Western blotting and qRT-PCR in paired LIHC tissues and adjacent normal tissues. (**H**) The immunofluorescence images showed the distribution of CUL2 in the A-431, U-251MG, and PC-3 cell lines. (**I**) The protein-protein interaction (PPI) network presents the proteins interacting with CUL2. All data are presented as the mean ± SD of three independent experiments. *p < 0.05, ** *p* < 0.01, *** *p* < 0.001, ns, no significance.

This study incorporated pan-cancer samples from publicly available databases for further investigation into various aspects, such as disparities in gene expression, the distribution of CUL2 mutations, the association between gene expression and survival rates, and immune infiltration. The study’s design process of this study is presented visually (Figure 1). First, we investigated the expression patterns of CUL2 in various organs and cell types. The results showed that the expression of CUL2 varied greatly across different organs and tissues (Figure 2A). However, the levels of CUL2 were consistently high across tumor cell lines (Figure 2B). Furthermore, by combining the TCGA and GTEx databases, we observed the expression levels of CUL2 in various types of tumor tissues. The results showed that CUL2 mRNA levels were highly expressed in many types of tumor tissues, including ACC, BLCA, BRCA, CESC, CHOL, COAD, ESCA, GBM, HNSC, LGG, LIHC, LUAD, LUSC, OV, PAAD, PRAD, SKCM, STAD, THCA, and UCS. In contrast, the mRNA level of CUL2 was low in LAML tissues compared to normal tissues (Figure 2C). We found that the protein expression level of CUL2 was significantly increased compared to that in normal tissues by using the CPTAC database (Figure 2D), which is consistent with the immunohistochemistry results (Figure 2E). To validate the results of bioinformatics analysis, we performed Western blotting and qRT-PCR experiments on tissue samples from patients diagnosed with HCC by clinical pathology. The results showed that compared to adjacent nontumor tissues, the protein and mRNA levels of CUL2 were significantly increased in HCC tissues (Figure 2F, 2G). Next, we analyzed immunofluorescence images of different cell lines to determine the subcellular distribution of the CUL2 protein. The results showed that CUL2 is mainly expressed in the nucleus of A-431, U-251MG, and PC-3 cells (Figure 2H). GeneMANIA is a web-based tool for predicting gene function and analyzing gene lists. It integrates information from various sources, such as protein-protein interactions, coexpression, pathways, and genetic interactions, to generate a functional interaction network for a set of genes. Finally, we developed a protein-protein exchange (PPI) network to discover possible biological associations of CUL2 (https://genemania.org/search/homo-sapiens/CUL2//) (Figure 2I). A network analysis of gene-disease interactions identified several functional partners of CUL2 linked to the integumentary system, genetic, familial or congenital, immune system, endocrine system, urinary system, and gastrointestinal diseases (Supplementary Figure 1). Based on the above findings, it can be inferred that CUL2 exhibits varying expression levels in different types of cancers, implying that it may significantly drive cancer progression.

### Genetic alterations of CUL2 across cancers

**Figure 3:**
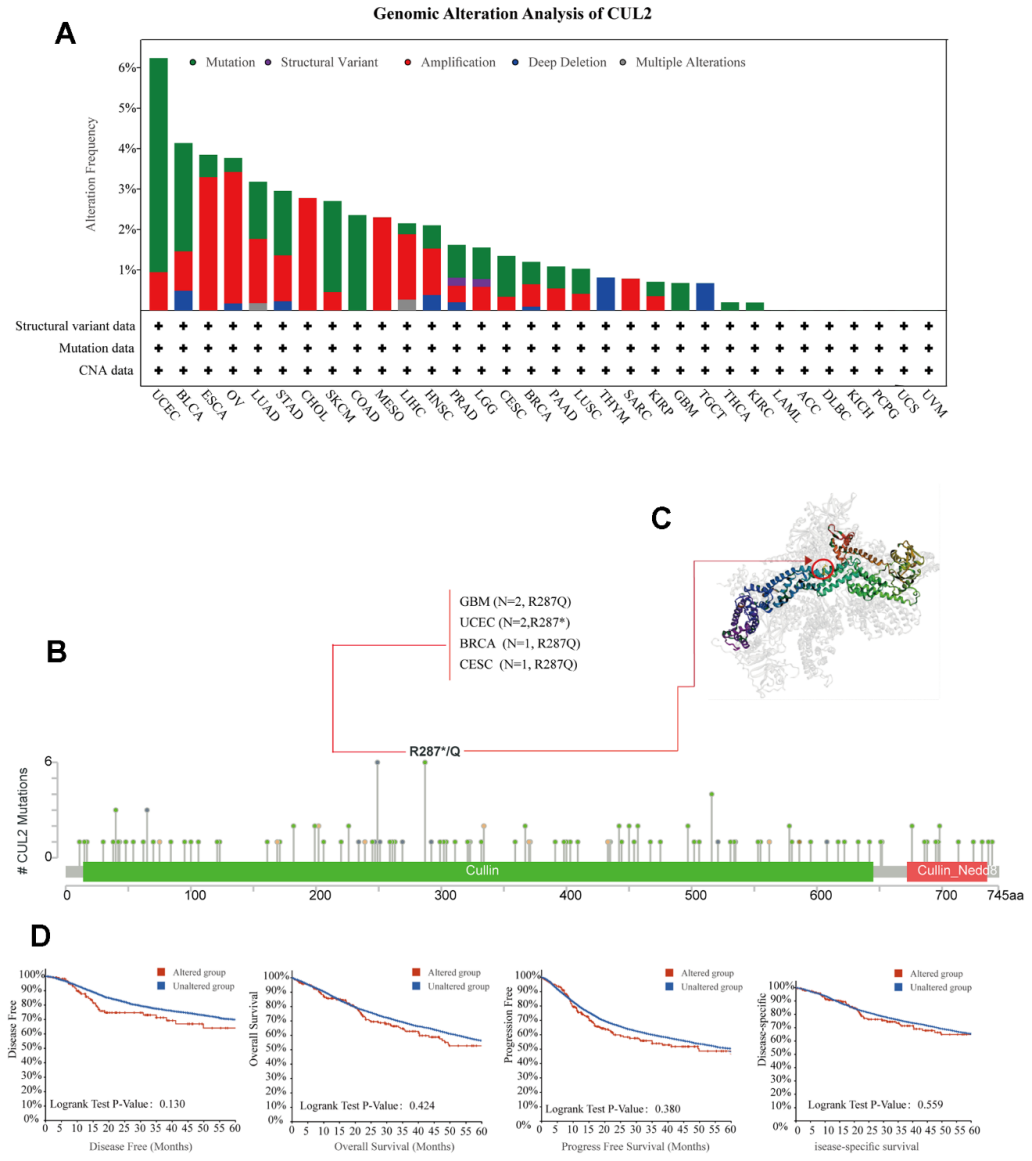
**Analysis of mutation feature of CUL2 in different tumors.** (**A**) The alteration frequency with mutation type in the CUL2 gene. (**B**) The specific alteration site of the CUL2 gene. (**C**) The 3D structure of CUL2 in the mutation site with the highest alteration frequency (R287*/Q) was displayed. (**D**) Survival analysis of patients with and without CUL2 alteration.

By using the cBioPortal tool on data from various cancers within the TCGA cohorts, we found that the highest alteration frequency (>6%) of CUL2 occurred. “Amplification” was the primary type of alteration, followed by “mutation”. Importantly, in cases of COAD, GBM, THCA, and KIRC, the sole type of CUL2 gene alteration observed was “mutation”. Amplification was the only mutation type in patients with MESO, CHOL, and SARC (Figure 3A). Additionally, the mutation count of CUL2 in different cancers is shown in Figure 3B. Furthermore, we also presented the types, sites, and case numbers of CUL2 genetic alterations. The mutation rate at position R287*/Q is the highest, and this mutation mainly occurs in patients with GBM, UCEC, BRCA, and CESC. The 3D structure of this mutation is shown in Figure 3C. It is worth noting that patients with CUL2 gene mutations have a poorer prognosis than patients without gene mutations; however, this difference has not yet reached statistical significance (Figure 3D).

### Correlation of CUL2 expression with prognosis

**Figure 4:**
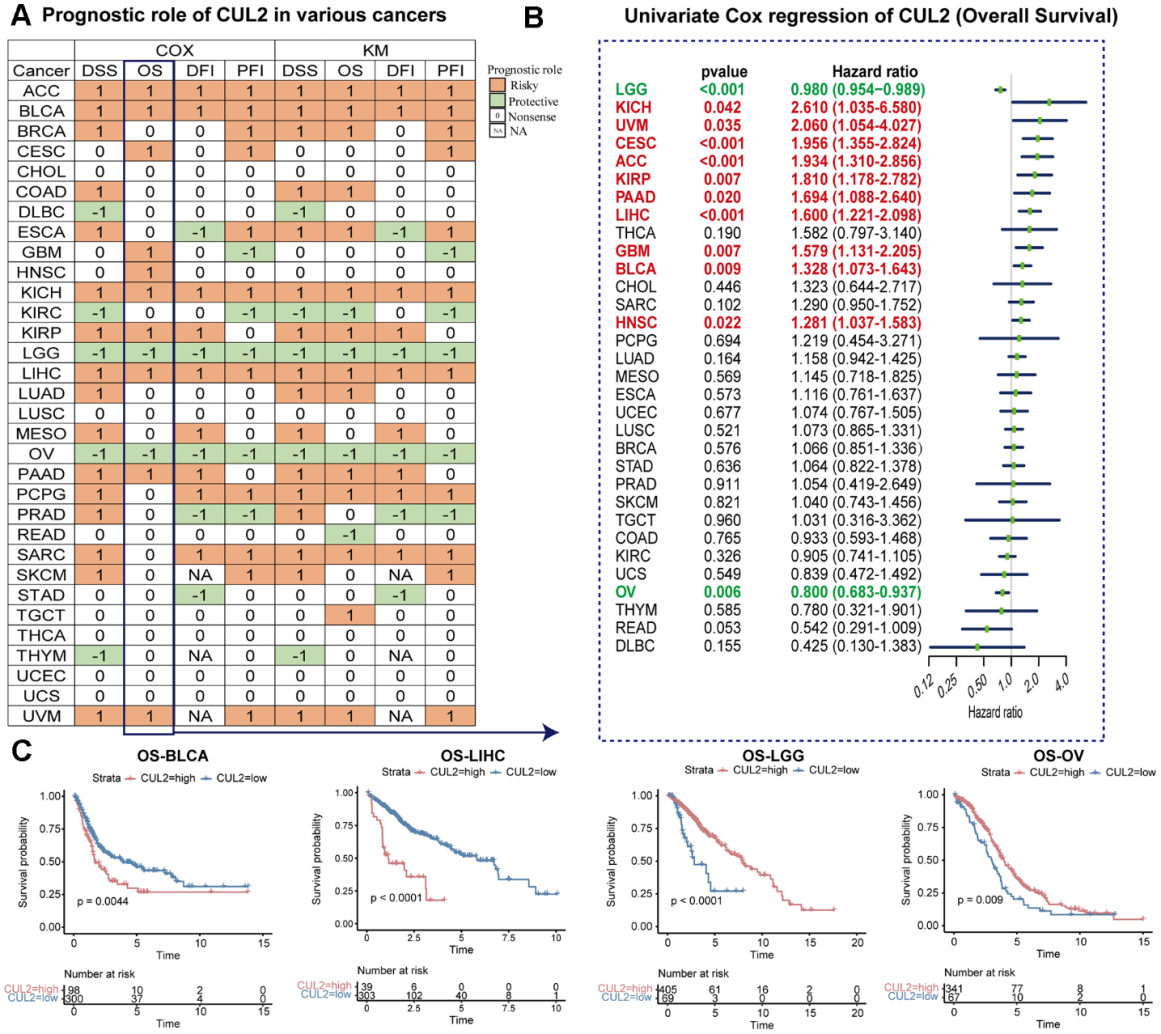
**Prognostic value of *CUL2* gene.** (**A**) This statement summarizes the relationship between the expression of CUL2 and cancer patient prognosis, specifically overall survival (OS), disease-specific survival (DSS), disease-free interval (DFI), and progression-free interval (PFI). Results were obtained using univariate Cox regression and Kaplan-Meier models, and only p-values < 0.05 are shown. The color red indicates that the high expression of CUL2 is a risk factor for poor prognosis. In contrast, green indicates that the high expression of CUL2 is a protective factor. (**B**) Univariate Cox regression analysis of CUL2 in pan-cancer (OS). (**C**) Kaplan-Meier overall survival curves of CUL2 in BLCA, LIHC, LGG, and OV.

To further investigate whether CUL2 can predict the prognosis of cancer patients, we explored the prognostic role of CUL2. Figure 4A summarizes the clinical predictive outcome patterns observed in the tested pan-cancer cohort, including OS, DSS, DFI, and PFI as indicators of prognosis. The OS analysis results suggested that CUL2 was harmful to patients with ACC, BLCA, BRCA, COAD, ESCA, KICH, KIRP, LIHC, LUAD, PAAD, PCPG, SARC, TGCT, and UVM, but it was a protective factor for patients with KIRC, LGG, OV, and READ. Given that OS included numerous noncancer deaths in its survival outcome endpoints, we conducted a DSS analysis instead, which is more pertinent to evaluating the effectiveness of cancer treatment. The OS analysis indicated that CUL2 is a prognostic risk factor for the cancers mentioned above, and intriguingly, the DSS analysis findings highly agreed with those from the OS analysis. We also examined the outcomes of DFI and PFI to provide additional evidence that CUL2 is a risk factor for most cancer types. Furthermore, the OS, DSS, PFI, and DSS outcomes showed that CUL2 was a protective factor for LGG and OV. In addition, the results shown in the forest plot suggested that the downregulation of CUL2 expression has special relationships with OS time prolongation in KICH (HR = 2.610 [95%CI, 1.035 to 6.580], p = 0.042), UVM (HR = 2.060 [95%CI, 1.054 to 4.027], p = 0.035), CESC (HR = 1.956 [95%CI, 1.355 to 2.824], p < 0.001), ACC (HR = 1.934 [95%CI, 1.310 to 2.856], p < 0.001), KIRP (HR = 1.810 [95%CI, 1.178 to 2.782], p = 0.007), PAAD (HR = 1.694 [95%CI, 1.088 to 2.640], p = 0.020), LIHC (HR = 1.600 [95%CI, 1.221 to 2.098], p < 0.001), GBM (HR = 1.579 [95%CI, 1.131 to 2.205], p = 0.007), BLCA (HR = 1.328 [95%CI, 1.073 to 1.643], p = 0.009), and HNSC (HR = 1.281 [95%CI, 1.037 to 1.583], p = 0.022). The upregulation of CUL2 expression was related to the time delay of OS: LGG (HR = 0.980 [95%CI, 0.954 to 0.989], p < 0.001) and OV (HR = 0.800 [95%CI, 0.683 to 0.937], p = 0.006) (Figure 4B). Several studies have indicated that CUL2 is closely related to the progression and prognosis of LIHC [8], and our results also suggested that a lower expression of CUL2 was related to a better survival outcome, indicating that CUL2 was a prognostic biomarker of OS in LIHC and BLCA. Furthermore, we also noted that elevated expression levels of CUL2 in LGG and OV were associated with favorable prognoses (Figure 4C). The prognostic significance of CUL2 varies across different types of cancer, with complex and multifaceted roles. Future research efforts should concentrate on elucidating the specific functions of the CUL2 protein within cancer cells.

### Pan-cancer gene set enrichment analysis (GSEA)

**Figure 5:**
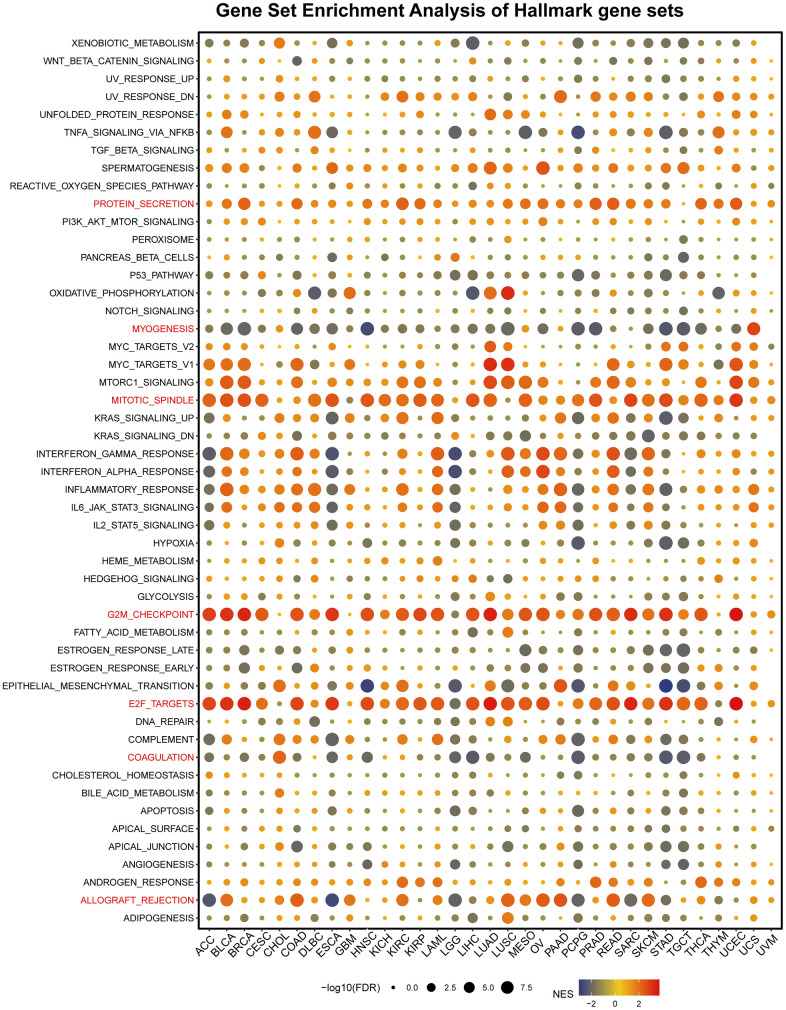
**Gene set enrichment analysis of hallmark gene sets.** The statement explains that in a certain analysis, circles were used to represent enriching terms in different types of cancer. The size of each circle indicated the false discovery rate (FDR) value of that term in that cancer. Additionally, the color of each circle represented the normalized enrichment score (NES) of that term in that cancer.

Next, our study analyzed a hallmark gene set composed of marker genes that define tumor physical status and progression. Specifically, we screened for differentially expressed genes between high- and low-CUL2 subgroups. Figure 5 shows that CUL2 expression was significantly related to some pathways, including protein secretion, myogenesis, mitotic spindle, G2/M checkpoint, E2F targets, coagulation, and allograft rejection. Importantly, CUL2 is negatively associated with myogenesis in most tumors, especially in BLCA, BRCA, COAD, HNSC, LUSC, PCPG, PRAD, STAD, and TGCT. In addition, CUL2 is positively associated with G2M checkpoint, E2F targets in all cancers, except for CHOL, DLBC, GBM, LGG, PAAD, THYM, UCS, and UVM. In LGG, CUL2 negatively correlates with many signaling pathways, including interferon-gamma/alpha response, inflammatory response, IL6-JAK-STAT3 signaling, IL2-STAT5 signaling, hypoxia, epithelial-mesenchymal-transition, coagulation, and allograft rejection. CUL2 is negatively correlated with EMT in HNSC, LGG, PCPG, STAD, and TGCT and positively associated with EMT in CHOL and PAAD. The prevention and treatment of metastasis are crucial for improving clinical outcomes as it is the leading cause of cancer-related deaths. Carcinogenesis has been linked to the process of epithelial-mesenchymal transition, a developmental program that enhances the mobility, invasion, and resistance to apoptosis of cancer cells, thereby promoting their ability to metastasize [26]. Therefore, the involvement of CUL2 in EMT suggests that CUL2 may have a crucial role in the development and oncogenesis of various cancers. These findings open avenues for further research into how CUL2 contributes to cancer establishment and progression.

### CUL2 expression is correlated with immune infiltration levels in multiple cancers

**Figure 6:**
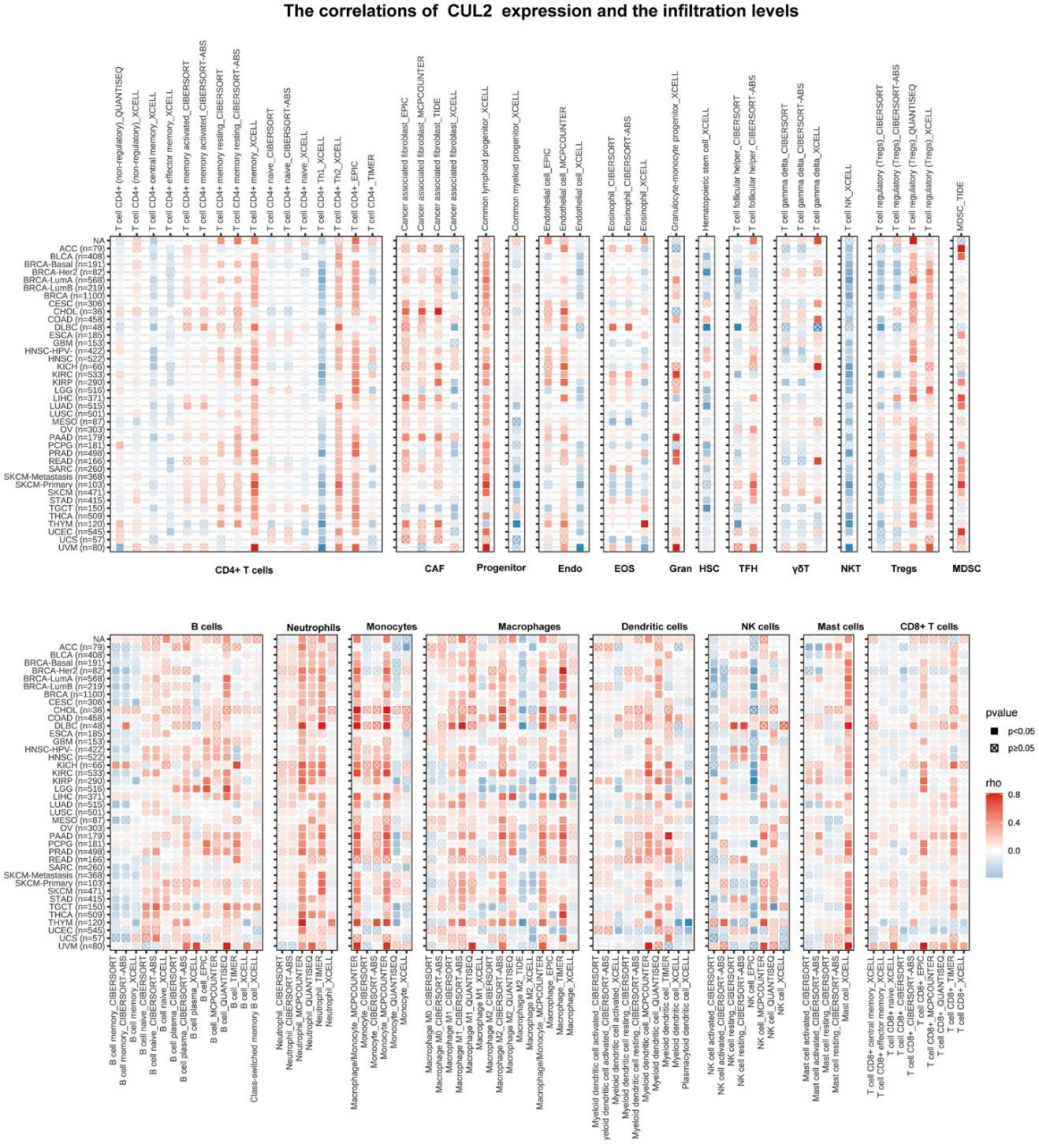
**Immune cell infiltration analyses.** This statement describes the association between CUL2 expression and the levels of different immune cell types in cancer. The immune cell types included CD4+ T cells, cancer-associated fibroblasts (CAF), progenitor cells, endothelial cells (Endo), eosinophils (Eos), hematopoietic stem cells (HSC), T follicular helper cells (Tfh), gamma-delta T cells (gdT), natural killer T cells (NKT), regulatory T cells (Tregs), B cells, neutrophils, monocytes, macrophages, dendritic cells, NK cells, mast cells, and CD8+ T cells. The results were represented by colors, with a positive correlation shown in red and a negative correlation shown in blue.

Given the close association between CUL2 expression and the immune pathway, we aimed to further investigate the potential relationship between CUL2 expression and immune cell infiltration. The findings indicate the presence of multiple types of immune and nonimmune cells within tumors, including CD4+ T cells, cancer-associated fibroblasts (CAFs), lymphoid and myeloid progenitor cells, endothelial cells (Endo), eosinophils (Eos), hematopoietic stem cells (HSCs), T follicular helper cells (Tfh), gamma delta T cells (γ/δT), natural killer T cells (NKT), regulatory T cells (Tregs), myeloid-derived suppressor cells (MDSCs), neutrophils, monocytes, B cells, dendritic cells, macrophages, mast cells, NK cells, and CD8+ T cells, across various types of cancers. As shown in Figure 6, CUL2 was positively associated with the infiltration levels of neutrophils, common lymphoid progenitors, and monocytes. CUL2 was negatively correlated with NKT and HSC infiltration levels in many tumors. To further investigate the correlation between CUL2 expression and immune infiltration in various types of cancer, we incorporated ImmuneScore, EstimateScore, StromalScore, and neoantigens into our analysis. Our results revealed that CUL2 expression correlated with ImmuneScore, EstimateScore, and StromalScore in some cancers (Supplementary Figures 2–4). Additionally, CUL2 expression was positively correlated with neoantigens in LUAD and STAD, and it was negatively correlated with neoantigens in READ and COAD (Supplementary Figure 5). Our findings suggest that the involvement of CUL2 in interactions with immune cells could have significant implications for the development, prognosis, and treatment of cancer.

### The association between CUL2 expression and immune checkmate inhibitors biomarkers

**Figure 7:**
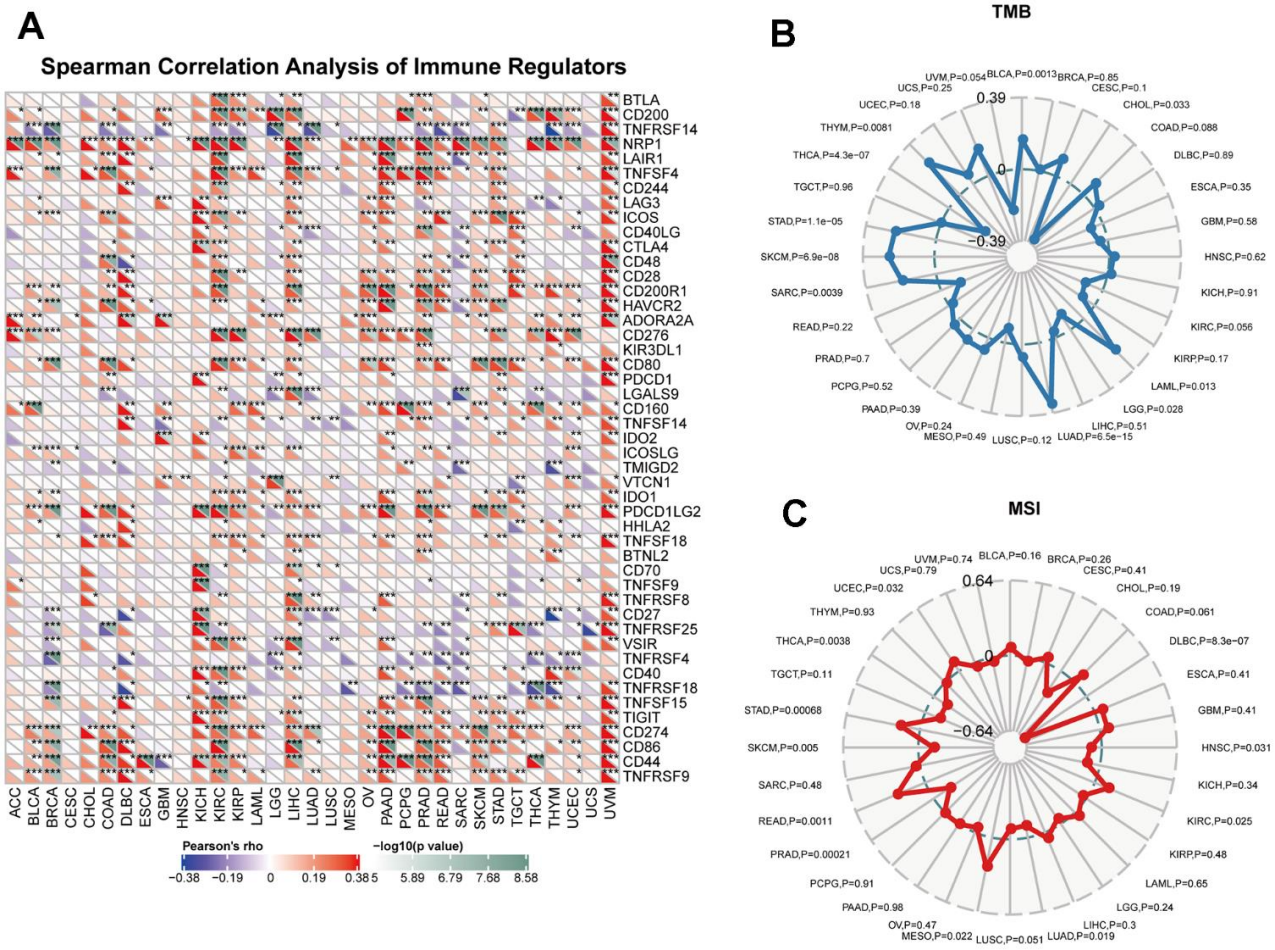
**Immunotherapy prediction analysis of CUL2 in the pan-cancer.** (**A**) the association between CUL2 expression and the expression of immune checkpoint genes in cancer. (**B**) Using a radar chart to visualize the correlation between CUL2 expression and TMB. (**C**) Using a radar chart to visualize the correlation between CUL2 expression and MSI. *p <0.05, ** *p* <0.01, *** *p* < 0.001.

Immune dysregulation plays a critical role in the development and progression of cancer, and identifying key immune regulators in pan-cancer can provide insight into potential therapeutic targets. We utilized a heatmap to display the correlation between CUL2 and tumor immune checkpoints in pan-cancer. As shown in Figure 7A, CUL2 positively correlated with NRP1, CD44, and PDCD1LG2 in most tumors. Besides, CUL2 showed a positive correlation with most immune checkpoints in KIRC, LIHC, PAAD, PRAD, STAD, and UVM. The goal was to obtain a better understanding of the role of CUL2 in predicting the efficacy of immune checkpoint inhibitors and to further evaluate the correlation between CUL2 expression and TMB and MSI. We found that CUL2 positively correlated with TMB in BLCA, THYM, STAD, SKCM, SARC, LUAD, and LAML but negatively correlated with TMB in THCA, LGG, and CHOL (Figure 7B). In addition, in the correlation analysis between CUL2 and MSI, CUL2 positively correlated with UCEC, STAD, READ, MESO, and KIRC. In contrast, it negatively correlated with THCA, SKCM, PRAD, HNSC, and DLBC (Figure 7C). Our findings suggest that CUL2 has the potential to be utilized as a predictor for the effectiveness of ICIs in their respective cancer types.

### Correlation between CUL2 and the regulation of MMR gene expression and DNA methylation in cancers

**Figure 8:**
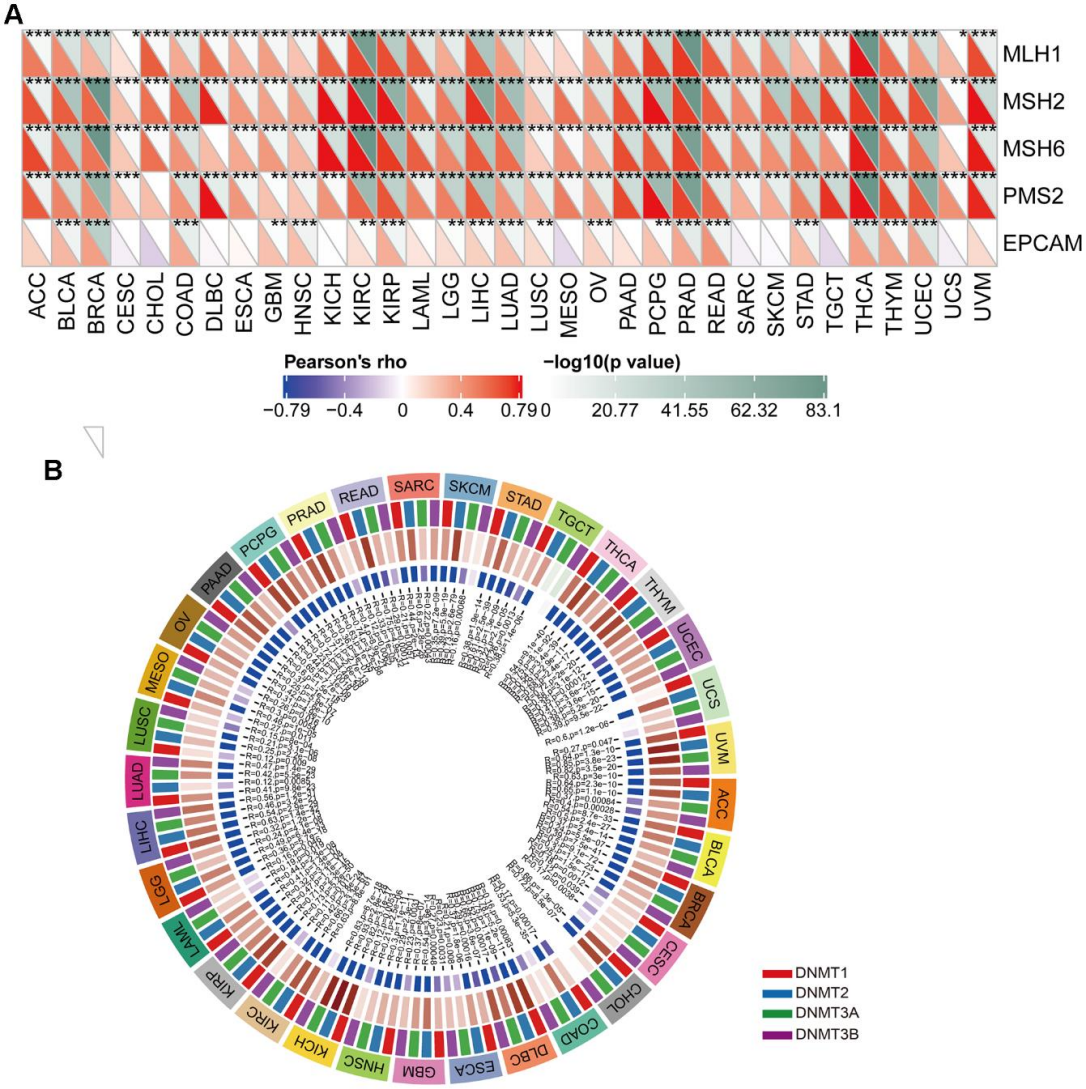
**The correlation analysis of CUL2 expression with MMR genes and DNA methyltransferases.** (**A**) The Spearman’s correlation analysis of CUL2 expression with MMR genes in cancers. (**B**) Spearman’s correlation to investigate the relationship between CUL2 expression and DNA methyltransferases in different types of cancer. *p < 0.05, ** *p* < 0.01, *** *p* < 0.001, ns, no significance.

MMR is a vital cellular mechanism that rectifies errors that arise during DNA replication, thus playing a crucial role in preserving the integrity of genetic material across all living organisms. Failure to correct these errors may result in mutations and genetic anomalies that have been implicated in various diseases, including cancer [27–29]. An analysis was conducted to evaluate the relationship between the expression levels of CUL2 and mutations in five MMR genes, namely MLH1, MSH2, MSH6, PMS2, and EPCAM, to determine the role of CUL2 in tumorigenesis. This study found that CUL2 was significantly associated with MLH1, MSH2, MSH6, PMS2, and EPCAM in most cancer types (Figure 8A). Another important factor that promotes tumorigenesis is abnormal DNA methylation, which has been touted as a promising biomarker for diagnosis, treatment, and prognosis [30]. The outcomes revealed a notable link between CUL2 and at least one of the four methyltransferase genes within the context of pan-carcinoma tissues (Figure 8B).

### CUL2 promoted HCC cells proliferation and invasion

**Figure 9:**
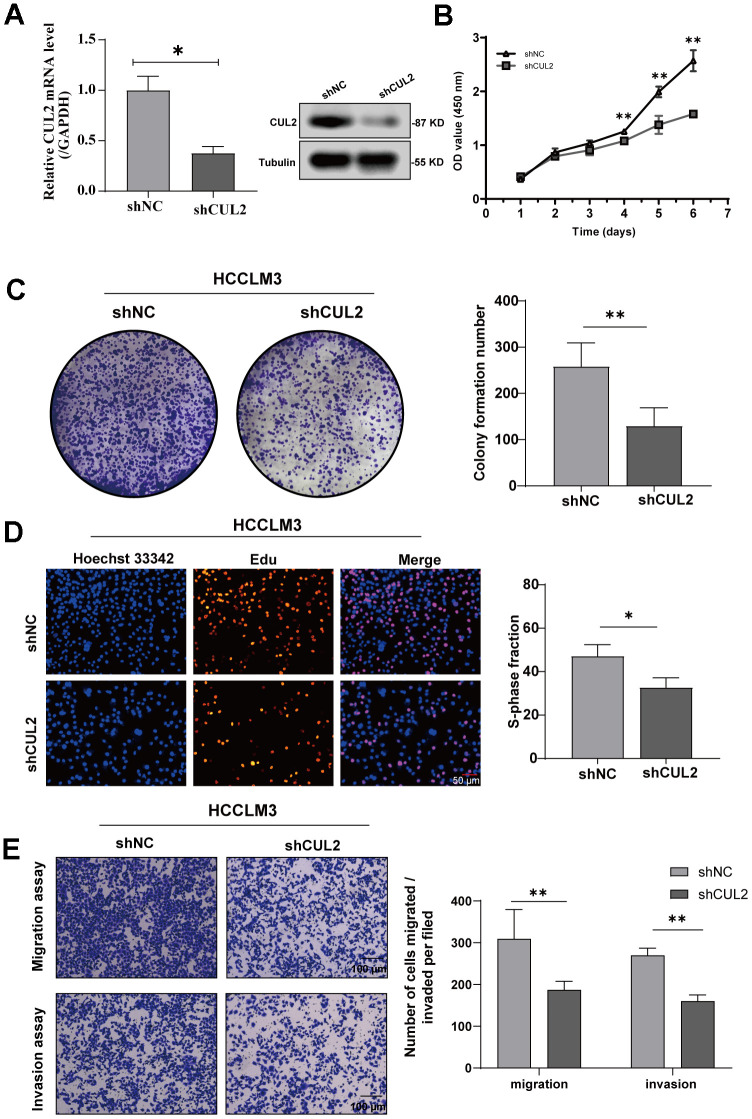
**Knockdown of CUL2 inhibits cell proliferation and invasion in HCCLM3 cells.** (**A**) The knockdown efficiency of shCUL2 was examined in HCCLM3 cells with western blotting, and qRT-PCR (**B**) Cellular growth curves were evaluated by CCK-8 assays in HCCLM3 cells. (**C**) Representative images and quantification of colony formation assays of HCCLM3 transfected with shCUL2. (**D**) Representative images and quantification of EdU assays to evaluate cell proliferation ability after transfecting shCUL2, magnification, ×200; scale bars, 50 μm. (**E**) Representative images and quantification of transwell assay to examine the invasion ability, magnification, ×200; scale bars, 100 μm. *p < 0.05, ** *p* < 0.01, *** *p* < 0.001, ns, no significance.

To reveal the role of CUL2 in the proliferation and invasion of HCC cells, we first reduced the expression of CUL2 by knocking down CUL2. The results showed that the protein and mRNA levels of CUL2 in the *shCUL2* group were significantly decreased compared with those in the *shNC* group (Figure 9A). Next, the results showed that the OD value (Figure 9B), the number of cell colony formations (Figure 9C), and the number of Edu-positive cells (Figure 9D) were significantly decreased in the *shCUL2* group, and transwell experiments show consistent results (Figure 9E).

## DISCUSSION

Immunotherapy has emerged as a promising and effective cancer treatment, highlighting the urgent need for identifying novel immune targets and biomarkers [31]. Our analysis of CUL2 transcriptome data through data mining revealed a significant increase in CUL2 RNA levels across nearly all tumor types, except for LAML, TGCT, KIRP, KIRC, READ, and KICH. In a pan-cancer cohort, the genetic analysis found that alterations in the CUL2 gene had a frequency of up to 6%, but the mutations observed were not specific. While the modifications did not show a statistically significant association with patient outcomes, it is suggested that they are unlikely to have a substantial role in the development of cancer.

We focused on investigating the clinical significance of CUL2 and its practical applications. We assessed the prognostic implications of CUL2 expression in various cancer types using Kaplan Meier and univariate Cox regression models. We evaluated the clinical outcomes using four indices: OS, DFI, DSS, and PFI, each reflecting prognosis under different conditions. Interestingly, we found that CUL2 expression was associated with increased risk and protection, suggesting a unique effect of CUL2 in each cancer type. CUL2 expression was found to correlate positively with OS in patients with KIRC, LGG, OV, and READ, suggesting a protective role in these cancers. However, in 11 out of 32 cancer types, including ACC, BLCA, BRCA, COAD, ESCA, KICH, KIRP, LIHC, LUAD, PAAD, PCPG, SARC, TGCT, and UVM, CUL2 expression was found to be a risk factor for poor prognosis. According to a detailed Kaplan-Meier survival curve analysis of patient data, high expression of CUL2 was found to be a risk factor for poor overall survival in patients with LIHC and BLCA. However, in LGG and OV, high CUL2 expression was associated with better clinical outcomes. These findings suggest that CUL2 may be a potent prognostic biomarker for many cancers.

Given the important implications of these findings, we proceeded to investigate the functional processes that could potentially involve CUL2. These pathways included G2M checkpoint, E2F targets, interferon-gamma/alpha response, inflammatory response, IL6-JAK-STAT3 signaling, IL2-STAT5 signaling, hypoxia, epithelial-mesenchymal-transition, coagulation, and allograft-rejection, all of which were highly significantly related. Research has demonstrated that stabilizing HIF by inhibiting CUL2 neddylation can protect mucosal inflammatory responses [32]. MicroRNA-574-3p Regulates HIF-α Isoforms Promoting Gastric Cancer Epithelial-Mesenchymal Transition via Targeting CUL2 [33]. Studies have shown that NLRC5 plays a role in limiting dengue virus infection by promoting the autophagic degradation of viral NS3 through the E3 ligase CUL2. This finding further highlights the critical involvement of CUL2 in the immune response [34]. IL6 and IL2 are well-established inflammatory factors that regulate tumor immunity by promoting lymphocyte growth and function. These interleukin-mediated signaling pathways have been shown to play an essential role in tumor immunology. They are actively being investigated as potential targets for cancer immunotherapy [35, 36]. However, the specific role of CUL2 in signaling pathways, such as immune regulation, remains to be further explored. Overall, our results suggest an immune-related mechanism, which prompted us to further explore the potential of CUL2 to predict patient responses to immunotherapy.

CUL2 plays a significant role in regulating antitumor immunity within the TME. Factors such as immune checkpoint proteins, TMB, and MSI can further influence immune response outcomes [37]. Improved patient results can be achieved by considering these factors and developing personalized cancer treatments targeting CUL2 and the immune system. Finally, in addition to the meticulous bioinformatics exploration, we have conducted experimental probes at the cellular level. Consistent with the bioinformatics results, WB and qRT-PCR results also confirmed that CUL2 expression was significantly upregulated in HCC tissues. In addition, *in vitro* cellular assays also concluded that reducing CUL2 inhibited the proliferation and invasive migration ability. In other types of tumors, CUL2 also plays an essential role in the development of cancer. CircSTX6 promotes the progression of pancreatic ductal adenocarcinoma by sponging miR-449b-5p and interacting with CUL2 [7]. In cervical cancer, CUL2 promotes development via mitogen-activated protein kinase signaling [38]. Epigenetic alterations, such as DNA methylation, are known to play a significant role in promoting cancer susceptibility and progression. Specifically, DNA hypomethylation has been shown to contribute to carcinogenesis and cancer development through various mechanisms [39, 40].

Our findings indicate a robust connection between the expression of CUL2 and the expression of these methyltransferases in the majority of cancer types, which suggests that CUL2 may play a role in cancer progression by influencing gene stability.

Although this study furnishes compelling evidence regarding CUL2’s predictive significance in clinical prognosis and potential implications for immunotherapy response across diverse cancer types, it is imperative to acknowledge certain limitations. Despite being traditionally viewed as a tumor-associated gene, CUL2 displayed diverse correlations with prognosis in the pan-cancer analysis. Additional experiments are needed to validate the proposed hypothesis. Although we have identified CUL2 as a potential predictor, it remains to be seen whether this approach can be practically applied in clinical settings. Moreover, this investigation focused on population-level analyses, neglecting individual differences that may affect treatment outcomes. Future research should address these issues to provide more personalized and effective cancer treatment. Our study employed a unique approach to conducting a comprehensive pan-cancer analysis and revealed that CUL2 expression is commonly dysregulated across many types of cancer.

## CONCLUSIONS

According to our research, the expression of CUL2 varies across different types of tumors and cells, with high levels of CUL2 being linked to poor survival rates and disease progression. We also observed a close correlation between CUL2 expression and immune infiltrating cell expression, immune checkpoint gene expression, TMB, MSI, MMR gene, and DNA methylation, among other factors. Our thorough analysis of these findings has revealed the significant immunological advantages of CUL2 as a biomarker for pan-cancer prognostics and immunotherapy. This study offers valuable insights for developing future immunotherapy and diagnostic studies, providing new treatment options for cancer patients.

## Supplementary Material

Supplementary Figures

Supplementary Tables
