# Supplementary material for: Comprehensive prognostic and immunological analysis of Cullin2 in pan-cancer and its identification in hepatocellular carcinoma
Source: Aging (Albany NY). 2024 May 22;16(10):8898–921. doi: 10.18632/aging.205848 (PMC11164483; doi:10.18632/aging.205848)
Supplement: Supplementary Figures [file aging-16-205848-s001.pdf]

SUPPLEMENTARY FIGURES

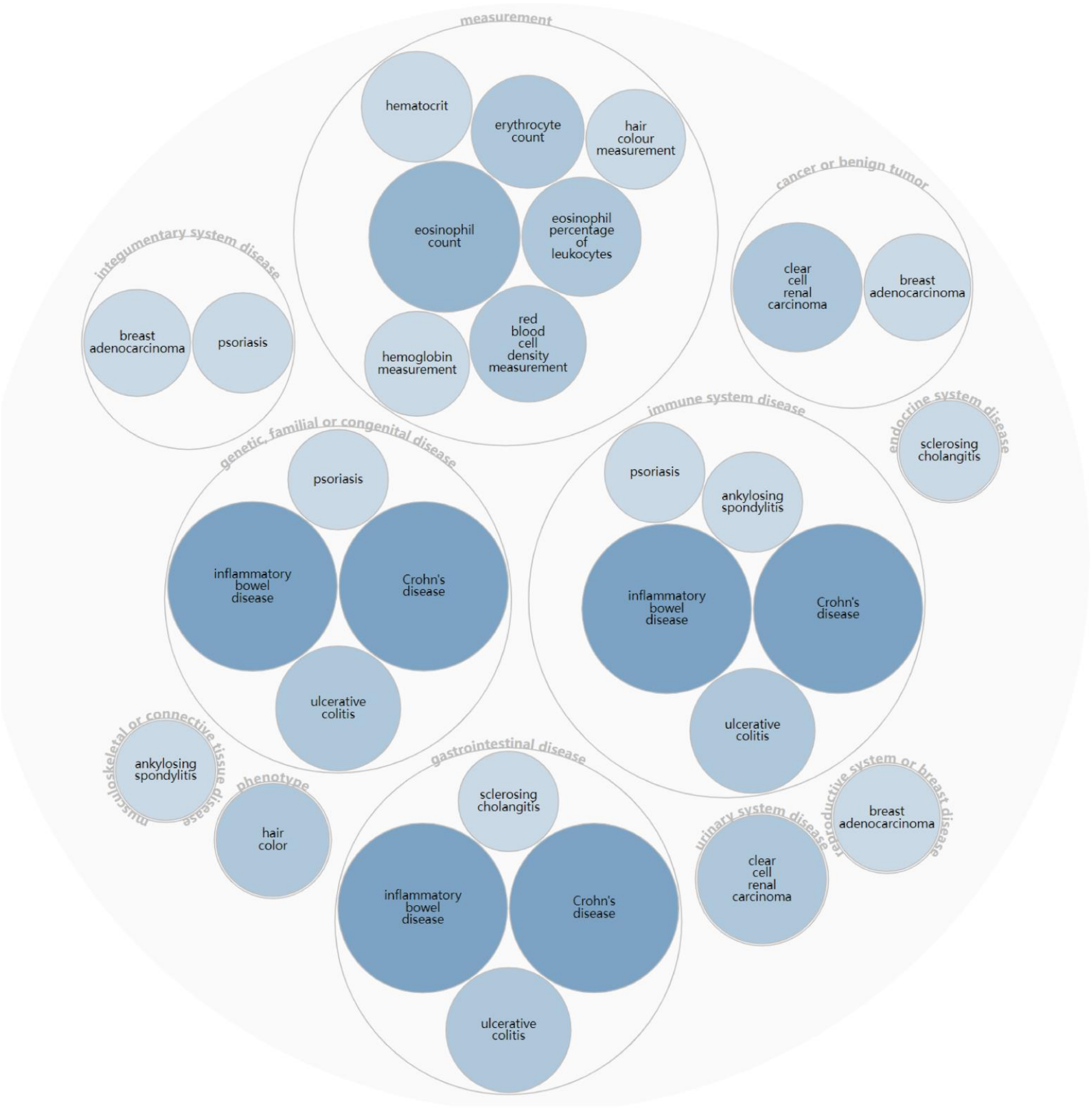

Supplementary Figure 1. The CUL2-associated disease network.

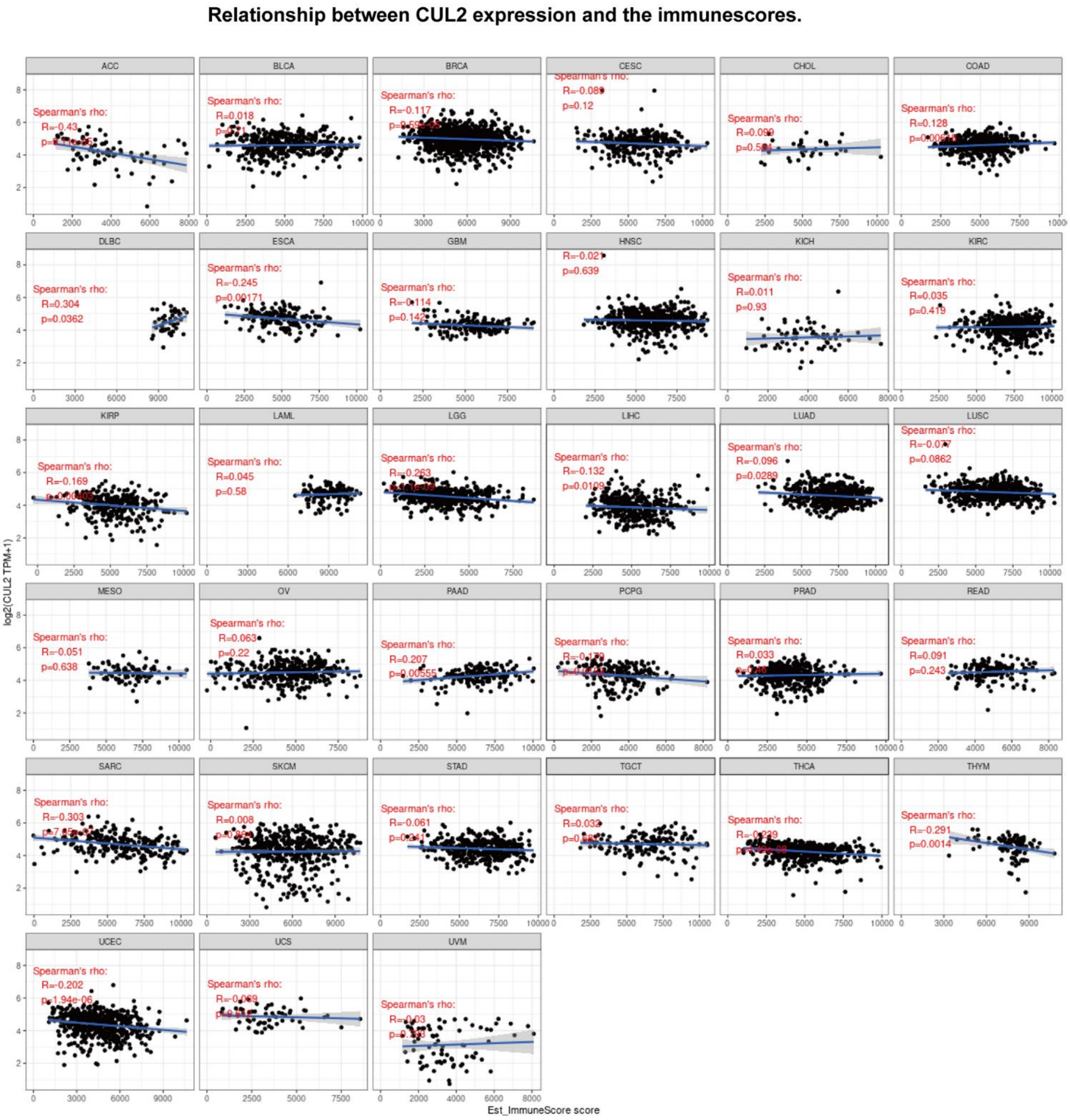

Supplementary Figure 2. Correlation analysis between expression levels of CUL2 and ImmuneScore in pan-cancer.

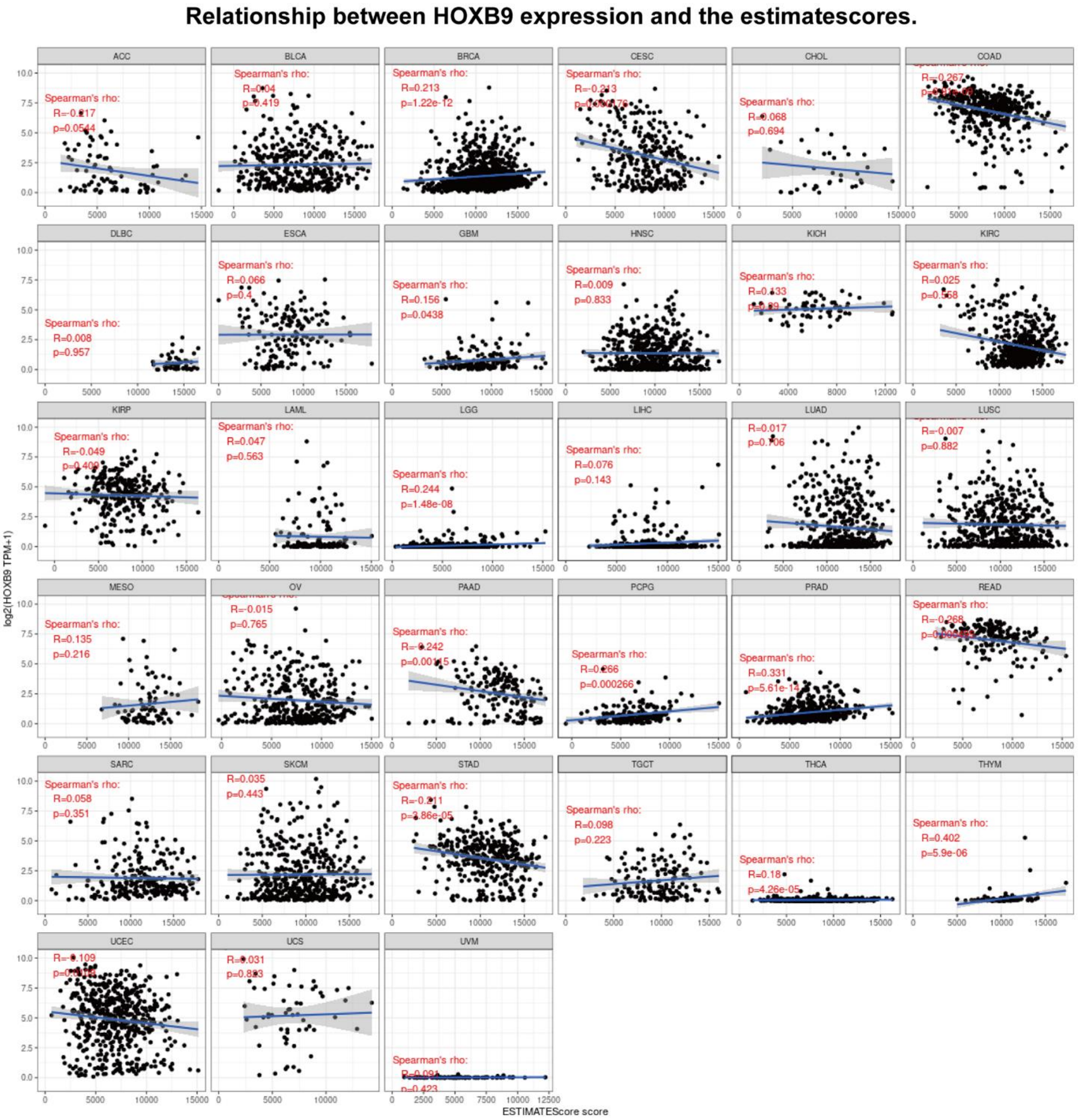

Supplementary Figure 3. Correlation analysis between expression levels of CUL2 and EstimateScore in pan-cancer.

Relationship between CUL2 expression and the stromalscores

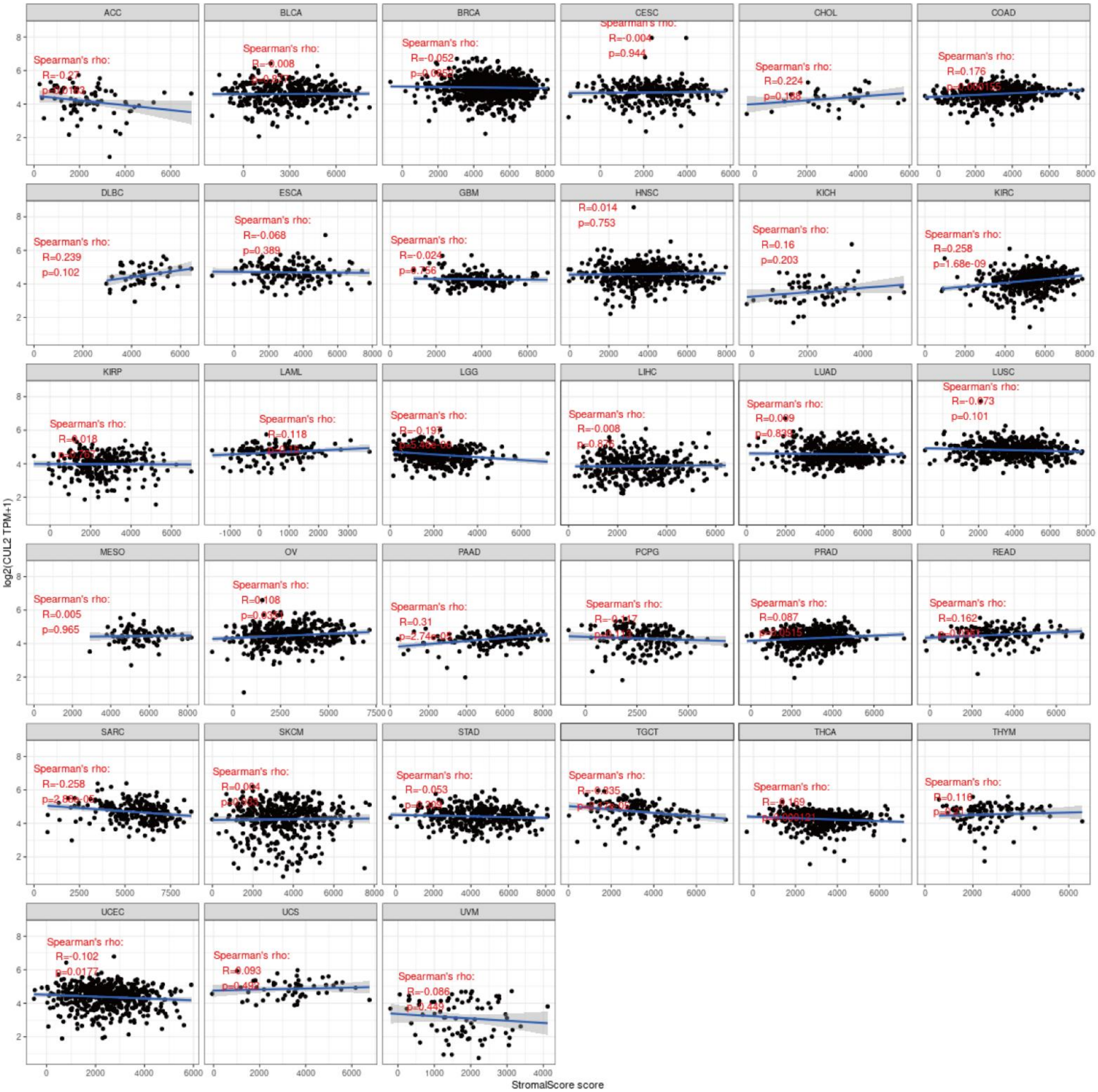

Supplementary Figure 4. Correlation analysis between expression levels of CUL2 and StromalScore in pan-cancer.

Relationship between CUL2 levels and neoantigens in pan-cancer

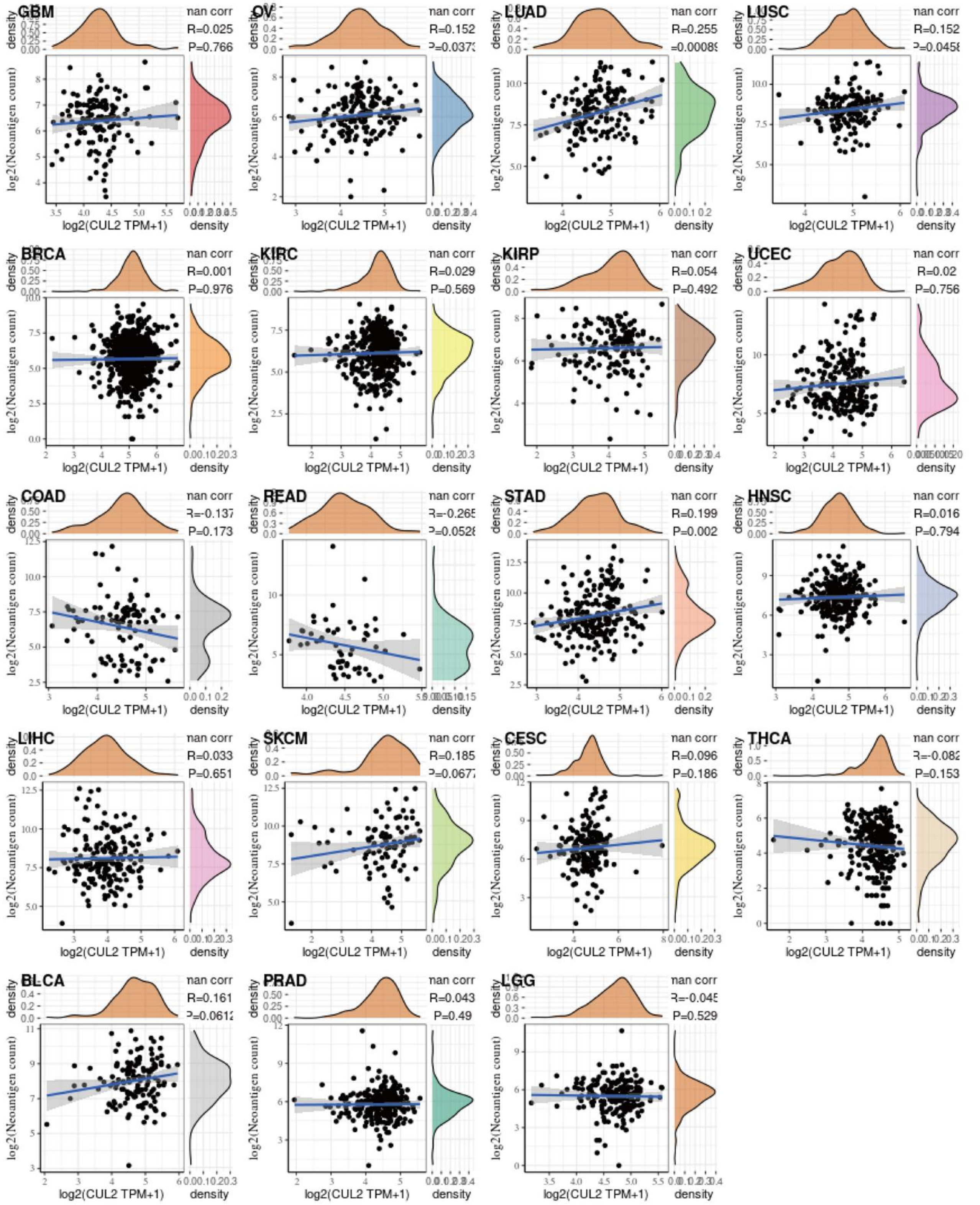

Supplementary Figure 5. Correlation analysis between CUL2 expression in pan-cancer and the number of tumors neoantigens in pan-cancer.
