# Supplementary material for: Comprehensive prognostic and immunological analysis of Cullin2 in pan-cancer and its identification in hepatocellular carcinoma
Source: Aging (Albany NY). 2024 May 22;16(10):8898–921. doi: 10.18632/aging.205848 (PMC11164483; doi:10.18632/aging.205848)
Supplement: Supplementary Tables [file aging-16-205848-s002.pdf]

SUPPLEMENTARY TABLES

Supplementary Table 1. Abbreviations of various cancers.

Abbreviations	Full name
ACC	Adrenocortical carcinoma
BLCA	Bladder Urothelial Carcinoma
BRCA	Breast invasive carcinoma
CESC	Cervical squamous cell carcinoma and endocervical adenocarcinoma
CHOL	Cholangiocarcinoma
COAD	Colon adenocarcinoma
DLBC	Lymphoid Neoplasm Diffuse Large B-cell Lymphoma
ESCA	Esophageal carcinoma
FPPP	FFPE Pilot Phase II
GBM	Glioblastoma multiforme
GBMLGG	Glioma
HNSC	Head and Neck squamous cell carcinoma
KICH	Kidney Chromophobe
KIPAN	Pan-kidney cohort (KICH+KIRC+KIRP)
KIRC	Kidney renal clear cell carcinoma
KIRP	Kidney renal papillary cell carcinoma
LAML	Acute Myeloid Leukemia
LGG	Brain Lower Grade Glioma
LIHC	Liver hepatocellular carcinoma
LUAD	Lung adenocarcinoma
LUSC	Lung squamous cell carcinoma
MESO	Mesothelioma
OV	Ovarian serous cystadenocarcinoma
PAAD	Pancreatic adenocarcinoma
PCPG	Pheochromocytoma and Paraganglioma
PRAD	Prostate adenocarcinoma
READ	Rectum adenocarcinoma
SARC	Sarcoma
STAD	Stomach adenocarcinoma
SKCM	Skin Cutaneous Melanoma
STES	Stomach and Esophageal carcinoma
TGCT	Testicular Germ Cell Tumors
THCA	Thyroid carcinoma
THYM	Thymoma
UCEC	Uterine Corpus Endometrial Carcinoma
UCS	Uterine Carcinosarcoma
UVM	Uveal Melanoma
OS	Osteosarcoma
ALL	Acute Lymphoblastic Leukemia

Supplementary Table 2. The antibodies and reagents used.

Reagent	Source	Identifier
BCA protein assay kit	Beyotime	Cat#P0012
Rabbit Anti-Cullin2	Abcam	Cat# ab-166917
Alpha Tubulin Monoclonal antibody	Proteintech	Cat# 66031-1-Ig
DAPI	Sigma-Aldrich	Cat# D9542
Crystal violet	Solarbio	Cat# G1062
Fetal bovine serum	Gibco	Cat# 10099141
PBS	Solarbio	Cat# P1020
Cell Counting Kit-8 Kit	Beyotime Institute of Biotechnology	Cat#C0037
Edu Kit	Ribobio	Cat#C10310
